# RETRACTION: Repression of CRNDE Enhances the Anti‐Tumour Activity of CD8 + T Cells Against Oral Squamous Cell Carcinoma Through Regulating Mir‐545‐5p and TIM‐3

**DOI:** 10.1111/jcmm.18566

**Published:** 2024-08-07

## Abstract

**RETRACTION:** Y. Ai, S. Wu, H. Gao, H. Wei, Z. Tang, X. Li, and C. Zou, “Repression of CRNDE Enhances the Anti‐Tumour Activity of CD8+ T Cells Against Oral Squamous Cell Carcinoma Through Regulating Mir‐545‐5p and TIM‐3,” *Journal of Cellular and Molecular Medicine* 25, no. 23 (2021): 10857–10868, https://doi.org/10.1111/jcmm.16909.

The above article, published online on 02 November 2021 in Wiley Online Library (wileyonlinelibrary.com), has been retracted by agreement between the journal Editor‐in‐Chief, Stefan Constantinescu; The Foundation for Cellular and Molecular Medicine; and John Wiley and Sons Ltd. The retraction has been agreed following an investigation into concerns raised by a third party, which revealed inappropriate duplications of image panels (Figures 4C,D, 6B and 7B) between this and other articles that were previously published in a different scientific context. The authors were unable to provide a satisfactory explanation, and the provided raw data could not explain the identified issues. Thus, the editors consider the conclusions of this manuscript substantially compromised. The authors were informed of the decision to retract but did not respond to the retraction decision or the wording.

**RETRACTION:** Y. Ai, S. Wu, H. Gao, H. Wei, Z. Tang, X. Li, and C. Zou, “Repression of CRNDE Enhances the Anti‐Tumour Activity of CD8+ T Cells Against Oral Squamous Cell Carcinoma Through Regulating Mir‐545‐5p and TIM‐3,” *Journal of Cellular and Molecular Medicine* 25, no. 23 (2021): 10857–10868, https://doi.org/10.1111/jcmm.16909.

The above article, published online on 02 November 2021 in Wiley Online Library (wileyonlinelibrary.com), has been retracted by agreement between the journal Editor‐in‐Chief, Stefan Constantinescu; The Foundation for Cellular and Molecular Medicine; and John Wiley and Sons Ltd. The retraction has been agreed following an investigation into concerns raised by a third party, which revealed inappropriate duplications of image panels (Figures 4C,D, 6B and 7B) between this and other articles that were previously published in a different scientific context. The authors were unable to provide a satisfactory explanation, and the provided raw data could not explain the identified issues. Thus, the editors consider the conclusions of this manuscript substantially compromised. The authors were informed of the decision to retract but did not respond to the retraction decision or the wording.

